# Physical activity improves mental health through resilience in Hong Kong Chinese adolescents

**DOI:** 10.1186/s12887-015-0365-0

**Published:** 2015-04-22

**Authors:** Frederick Ka Wing Ho, Lobo Hung Tak Louie, Chun Bong Chow, Wilfred Hing Sang Wong, Patrick Ip

**Affiliations:** Department of Paediatrics and Adolescent Medicine, The University of Hong Kong, Hong Kong, China; Department of Physical Education, Hong Kong Baptist University, Hong Kong, China; Queen Mary Hospital, 1/F, New Clinical Building, Hong Kong, China

## Abstract

**Background:**

Adolescent mental health problems are global public health concern. Primary prevention through physical activity (PA) has been suggested as a potential approach to tackling this problem. Studies in Western countries have provided some evidence of a relationship between PA and adolescent mental health, but the evidence in China is not sufficient. Furthermore, the mechanism behind this relationship has not been empirically tested. The present study aimed at testing the association between PA and mental well-being of Chinese adolescents and to investigate whether a psychological (self-efficacy and resilience) and social (school and family connectedness) mediation model is valid to explain such a relationship.

**Methods:**

A total of 775 Chinese students in Grades 7 and 8 were recruited in this cross-sectional study. The participants were given questionnaires to assess their PA level, mental well-being, and the potential mediators. Path models were used to analyse the association between PA and mental well-being, and the roles of potential mediators.

**Results:**

The PA level was significantly correlated with the adolescent’s mental well-being (r = 0.66, p < 0.001), self-efficacy (r = 0.21, p < 0.001), and resilience (r = 0.25, p < 0.001), but not with school connectedness (r = 0.05, p = 0.15) or family connectedness (r = 0.06, p = 0.13). After adjusting for potential confounders in the path model, the PA level was significantly associated with mental well-being (b = 0.52, p < 0.001), and resilience was the only significant mediator (b = 0.31, p < 0.001), which contributed to 60% of this relationship.

**Conclusions:**

There was a significant positive association between the PA level and mental well-being of Chinese adolescents. Resilience mediated the majority of this relationship. Promoting physical activities that build up resilience could be a promising way to improve adolescent mental health.

## Background

Adolescent mental health problems are a global public health concern with increasing disease burden [1]. Although adolescents are less likely to have clinical mental illnesses than adults, their mental well-being should not be overlooked because many adult mental disorders originate in childhood [2].

Mental disorders are estimated to affect 10% to 20% of children and adolescents worldwide, resulting in short- and long-term adverse consequences such as school disengagement, poor quality of life, morbidity, and even mortality [3]. The situation in Hong Kong is no less worrying. At least 16% of adolescents in Grades 7 to 9 had mental disorders according to the Diagnostic and Statistical Manual of Mental Disorders—4th Edition (DSM-IV) and an additional 22% had related symptoms. Among these mental illnesses, oppositional defiant disorder and anxiety disorder were the most common with a prevalence of 6.9% and 6.8%, respectively [4].

Conventional strategies for treating mental illnesses were based on the disease model, which focused on treating the disease. However, emerging evidence has suggested that primary prevention can improve the overall mental well-being of children and adolescents, even if they do not exhibit clinical symptoms [3].

Physical activity (PA) is one possible approach to implement primary prevention. Some rigorous studies have already shown PA has benefits on mental health. Randomised controlled trials have suggested exercise had benefits in reducing depression and anxiety symptoms [5], and a review reported a positive association between PA and self-esteem amongst children and adolescents [6]. However, there is also evidence against this positive relationship, particularly when mental health was conceptualised as internalising and externalising behaviours [7]. Therefore, it still remains unclear whether PA benefits children and adolescents’ overall mental well-being or whether the benefits are restricted to only a certain type of mental disorder. In addition, evidence derived from Western countries may not be applicable to other populations because mental health is highly dependent on culture, for example, there are different attitudes and values towards the diagnosis and treatment of mental illness [8]. Preliminary data from a Chinese school survey reported an inverse association between PA level and depression symptoms, but the study was only a cross-sectional survey concerned with a specific type of mental disorder. The findings were not sufficiently strong to draw a conclusion [9].

In addition to the inconclusive relationship between PA and mental well-being, the mechanisms involved in this relationship are also uncertain. Researchers have attempted to explain the relationship with biological, psychological, and social hypotheses. The biological hypothesis mainly focuses on neurotransmitters linked to PA such as serotonin, dopamine, and β-endorphin. The psychological hypothesis suggests that PA, as a type of challenge, could improve an individual’s confidence and skills such as self-efficacy and resilience, which in turn enhances their overall mental well-being and reduces the risk of mental illness [10,11]. The social hypothesis is based on the assumption that PA promotes social interactions and connectedness. Many forms of PA and exercise are social activities in which individuals can acquire social skills and strengthen their social network. The increase in social capital could provide support against stressors and improve their mental health [10,11]. However, psychological and social hypotheses have been rarely tested empirically [12].

In view of this, the present study aimed to: 1) investigate the relationship between PA and holistic mental well-being among Chinese adolescents with adjustment for potential confounders, 2) test whether the psychological (self-efficacy and resilience) and social (school and family connectedness) hypotheses for this relationship can be supported by empirical data, and 3) establish the relative importance of these factors.

## Methods

### Study design and participants

Students in Grades 7 and 8 (ages 12 to 14) from 12 secondary schools in Hong Kong, China were recruited into this cross-sectional study. Adolescents in Grades 7 and 8 were chosen because mental health problems often start to emerge in this age group [13]. Secondary schools with physical education teachers who graduated from a Physical Education Department at a local university were contacted and invited to join the study. Out of the 15 schools contacted, 12 schools agreed to support and participate in the study. The 12 participating schools were evenly distributed in terms of academic performance and were located in different socioeconomic areas of Hong Kong. The school neighbourhood median monthly household incomes ranged from USD 1,410 to USD 3,205 (median in Hong Kong = USD 2,210) and the neighbourhood proportion of public rental housing ranged from 0% to 75% (mean proportion in Hong Kong = 31%) [14].

Invitation and informed consent letters were sent to the students’ parents through the participating schools. After receiving parental consent, students were asked to complete a questionnaire in class with the support of a research assistant.

### Measurements

#### Mental well-being

The participant’s holistic mental well-being was assessed using the Mental Component Summary score (MCS-12) of the Chinese SF-12v2 Health Survey. The SF-12v2 is a 12-item self-report survey that measures the participant’s functional health over a 4-week recall period. The survey has been shown to be valid for Chinese adolescents in Hong Kong and the MCS-12 score can reliably distinguish adolescents with mental problems from the healthy population [15]. The MCS-12 was scored using the official computer program (QualityMetric Inc., USA).

#### Physical activity level

Physical Activity Rating Questionnaire for Children and Youth (PARCY) was used to assess the student’s physical activity level. The self-report questionnaire was developed based on the Jackson Activity Coding [16] and the Godin-Shepard Activity Questionnaire modified for adolescents [17]. The questionnaire consists of one item that evaluates the student’s average weekly physical activity level in the past year and takes into consideration the physical activity frequency, duration, and intensity. The PARCY score is assessed on 11-point scale ranging from 0 (‘*no exercise at all in the last year*’) to 10 (‘*doing vigorous exercise almost every day in the last year*’). This questionnaire was reported to have good criterion and convergent validity, and test-retest reliability in local studies on Chinese adolescents, and has been used in previous clinical epidemiological studies [18,19].

#### Potential mediators – psychological pathway

General self-efficacy is defined as a person’s strength of belief in his/her general capability. In this study, the psychological concept was measured using the unidimensional General Self-Efficacy Scale, which has 10 items scored on a 5-point Likert scale. The scale has been previously validated and used in the Chinese population [20].

Resilience is defined as the ability to recover from an adverse situation. In this study, it was measured using the Chinese version of Connor-Davidson Resilience Scale (CD-RISC) [21], which has 25 items scored on a 5-point Likert scale and aggregated into a single resilience score. Previous studies have shown that CD-RISC had good validity and reliability among Chinese adolescents [22].

#### Potential mediators – social pathway

Social connectedness of a person is how connected and close he/she is to a particular social group such as school or family. This concept was assessed by the Resnick School Connectedness Scale (RSC) and the Resnick Family Connectedness Scale (RFC), respectively [23]. The RSC has six items that are scored on a 5-point Likert scale, which measures the participant’s closeness to their school (e.g., ‘*feel part of your school*’). The RFS has 13 items that are scored on a 5-point Likert scale, which assesses the participant’s closeness to and perceived support from their family and parents (e.g., ‘*feeling loved and wanted within the family*’). Both scales have been shown to have acceptable reliability and validity in the Chinese population [24,23].

#### Socioeconomic status

The socioeconomic status (SES) composite score was constructed using principal component analysis of four neighbourhood sociodemographic statistics (median monthly household income, proportion of public rental housing, proportion of professional working population, and proportion of adults with post-secondary education). These statistics were extracted from a recent population by-census [14] and these method have been validated to be able to determine SES gradients within a population [25].

### Statistical analysis

**Figure 1:**
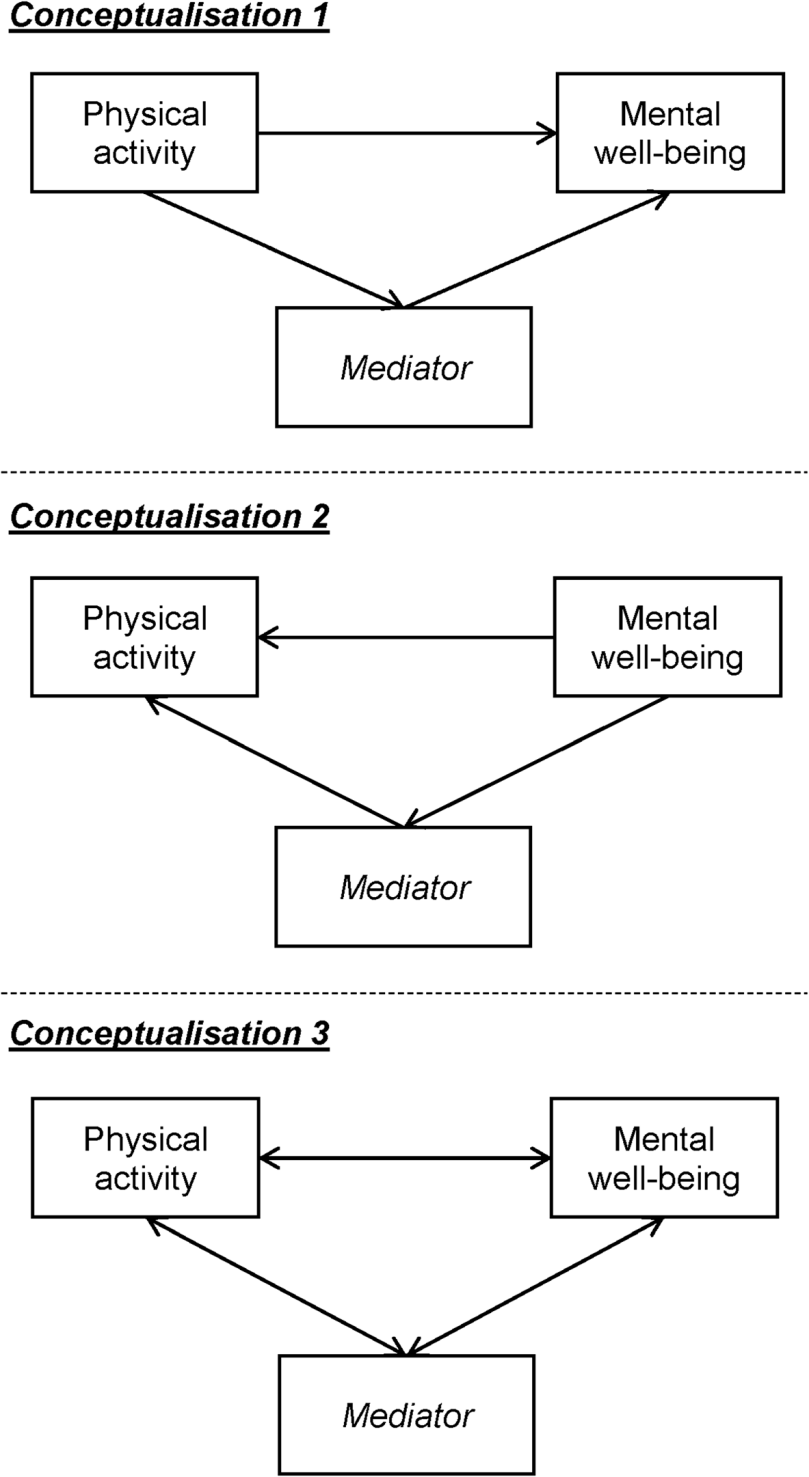
Structural diagram of the single-mediator path models in the three causal conceptualisations. Residuals and potential confounders (SES and gender) were omitted from the diagram for a clearer presentation.

Pearson’s product–moment correlation coefficients were used to examine crude bivariate associations. For each potential mediator, single-mediator path models in three causal conceptualisations (Figure 1) were considered. These models were compared using Akaike Information Criteria (AIC) and model fit indices. The model with the smallest AIC and best-fit indices was chosen for each potential mediator and was used to evaluate crude mediation effects and mediation proportions.

The significant mediators identified in the single-mediator models were entered into the multiple-mediator model to estimate their statistical significance and relative importance. A multi-group path model was used to compare differences in the associations between female and male adolescents.

The path models were estimated with maximum likelihood and Huber’s robust standard error estimator to account for the multilevel data structures [26]. A path model was considered to have satisfactory fit if its comparative fit index (CFI) was not smaller than 0.96, the root mean square error of approximation (RMSEA) was not larger than 0.06, and the standardised root mean square residual (SRMR) was not larger than 0.09 [27]. Full information maximum likelihood was used to handle missing data, which has been shown to be comparable to multiple imputations [28]. Sensitivity analyses were carried out using bootstrap standard error estimations and complete case analysis.

#### Power analysis

The full path model including PA level, mental well-being, self-efficacy, resilience, school connectedness, family connectedness, gender, and SES was assumed for the power analysis. The full model had eight degrees of freedom, which requires a sample size of 753 to detect an RMSEA close fit at 0.05 significance level and 80% statistical power [29]. A total of 775 subjects were included in this study, which should provide sufficient statistical power.

### Ethics statement

Written informed consent was obtained from all participants and their parents or guardians. The study was approved by the Ethical Committee/Institutional Review Board of The University of Hong Kong/Hospital Authority Hong Kong West Cluster.

## Results

### Participant characteristics

Among the 779 students from Grades 7 and 8 invited to the study, 775 (99.5%) agreed to participate. Descriptive statistics of their characteristics and key measurements are shown in Table 1. The average age of the participants was 12.28 years (SD = 0.77) and the female-to-male ratio was 1.26. The students’ mean PA level measured by PARCY was 5.41, corresponding to light daily PA for at least 20 minutes (excluding physical education lessons). The PARCY score for male students (6.12) was significantly higher than for female students (4.86, p < 0.001, unpaired t-test). The mean MCS-12 score was 48.02 (SD = 8.43) out of a maximum score of 100.

**Table 1:** **Descriptive statistics and Pearson’s correlation coefficients of the measurements**

				**Correlation coefficients**						
	**n**	**Mean**	**SD**	**2**	**3**	**4**	**5**	**6**	**7**	**8**
1. Age (years)	769	12.28	0.77	0.00	−0.02	0.02	0.02	−0.05	−0.07	0.06
2. Physical activity level	695	5.41	2.68		0.66***	0.21***	0.25***	0.05	0.06	0.09*
3. Mental well-being	751	48.02	8.43			0.33***	0.44***	0.34***	0.35***	0.02
4. Self-efficacy	757	27.70	6.06				0.66***	0.26***	0.25***	0.09*
5. Resilience	736	64.38	16.57					0.43***	0.39***	0.05
6. School connectedness	753	23.58	4.98						0.67***	−0.05
7. Family connectedness	754	43.28	9.47							−0.01
8. SES	775	−1.69	1.00							

n = Effective sample size; SD = Standard Deviation; SES = Socioeconomic status; *p < 0.05; ***p < 0.001.

### Correlations

Pearson’s product–moment correlation coefficients of the key measurements are shown in Table 1. Mental well-being was found to have significant associations with PA level (r = 0.66, p < 0.001), self-efficacy (r = 0.33, p < 0.001), resilience (r = 0.44, p < 0.001), school connectedness (r = 0.34, p < 0.001), and family connectedness (r = 0.35, p < 0.001). PA was found to be significantly correlated with self-efficacy (r = 0.21, p < 0.001) and resilience (r = 0.25, p < 0.001) but not with the two social variables.

### Single-mediator path models

Results of the single-mediator path model are shown in Table 2. Causal conceptualisation 1 (Figure 1) had the smallest AIC and best-fit indices for all potential mediators, which supported our initial causal assumptions. Without considering mutual influences, resilience appeared to be the most significant mediator (b = 0.35, p < 0.001, mediation proportion = 68%), which was followed by self-efficacy (b = 0.19, p < 0.001, mediation proportion = 36%), family connectedness (b = 0.08, p = 0.06, mediation proportion = 16%), and school connectedness (b = 0.07, p = 0.10, mediation proportion = 15%).

**Table 2:** **Results of the single-mediator path models and estimates of the mediated effects**

					**Mediated association between PA and mental well-being in the best fit model**				
**Models**	**AIC**	**CFI**	**RMSEA**	**SRMR**	**Coefficient estimates**	**95% CI lower band**	**95% CI upper band**	**p-value**	**Proportion of mediation**
***Self-efficacy as a mediator***									
**Conceptualisation 1**	**19464.12**	**0.969**	**0.055**	**0.020**	0.19	0.10	0.28	< 0.001	0.36
Conceptualisation 2	19539.32	0.965	0.059	0.022	-	-	-	-	-
Conceptualisation 3	19563.74	0.593	0.159	0.068	-	-	-	-	-
***Resilience as a mediator***									
**Conceptualisation 1**	**20737.04**	**0.980**	**0.054**	**0.020**	0.35	0.23	0.47	< 0.001	0.68
Conceptualisation 2	20737.81	0.977	0.057	0.022	-	-	-	-	-
Conceptualisation 3	20836.68	0.691	0.158	0.068	-	-	-	-	-
***School connectedness as a mediator***									
**Conceptualisation 1**	**19154.74**	**0.935**	**0.079**	**0.027**	0.07	−0.01	0.16	0.10	0.15
Conceptualisation 2	19155.31	0.933	0.080	0.028	-	-	-	-	-
Conceptualisation 3	19254.17	0.571	0.162	0.070	-	-	-	-	-
***Family connectedness as a mediator***									
**Conceptualisation 1**	**20131.63**	**0.931**	**0.082**	**0.028**	0.08	0.00	0.17	0.06	0.16
Conceptualisation 2	20132.22	0.929	0.083	0.029	-	-	-	-	-
Conceptualisation 3	20231.08	0.570	0.163	0.070	-	-	-	-	-

Bold entries indicate the chosen conceptualisation. CI = Confidence interval, AIC = Akaike Information Criteria, CFI = Comparative fit index, RMSEA = Root mean square error of approximation, SRMR = Standardised root mean square.

### Multiple-mediator path models

**Figure 2:**
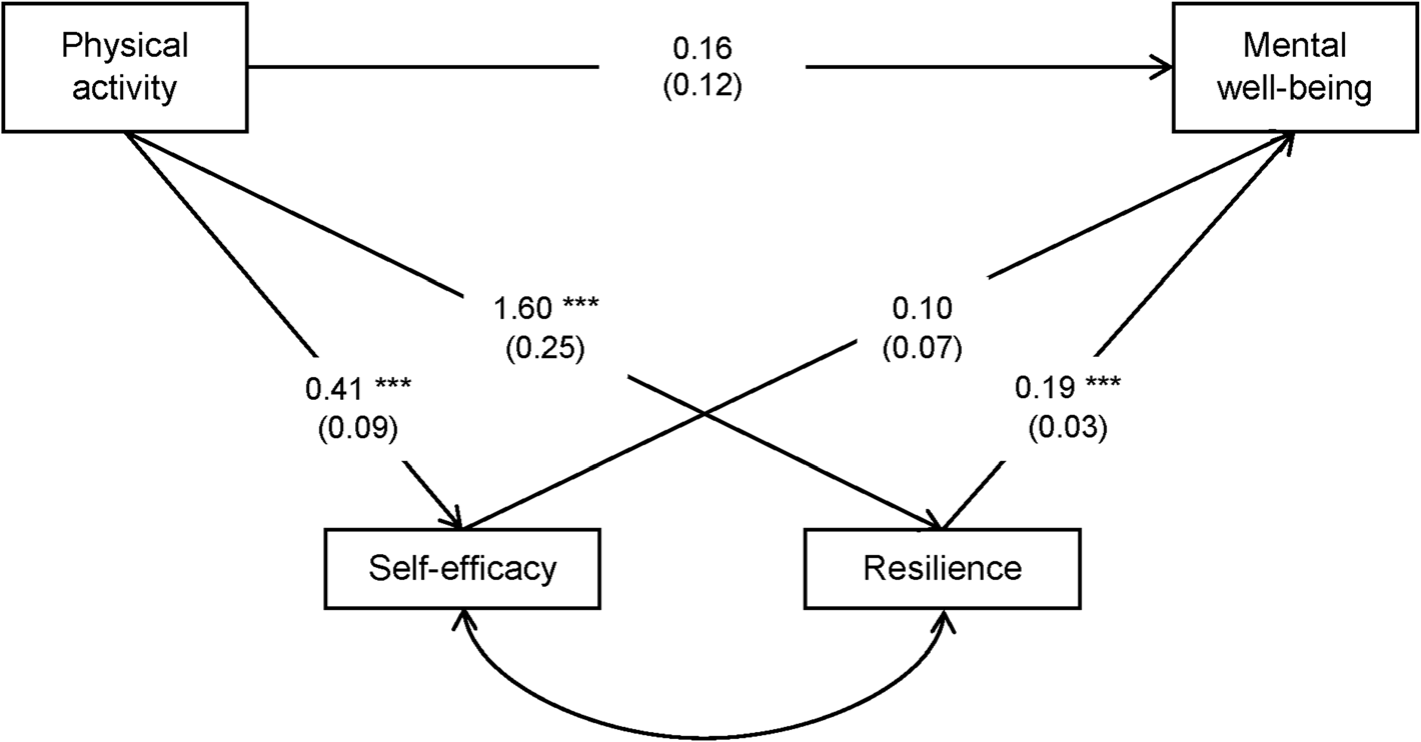
Structural diagram of the multiple-mediator path model. Residuals, covariance estimates and potential confounders (SES and gender) were omitted from the diagram for a clearer presentation. Coefficient estimates with standard errors in parentheses are shown on the paths.

A diagram of the multiple-mediator path model is shown in Figure 2. The model had satisfactory model fit. After adjusting for the two indirect pathways, the association between MCS-12 and PA level was no longer significant. PA was significantly associated with both self-efficacy and resilience, but only resilience was significantly associated with mental well-being. Thus, resilience was identified as the only significant mediator, which accounted for 60% of the total association. A detailed model summary can be found in Table 3.

**Table 3:** **Direct and indirect associations between PA level and mental well-being**

	**Coefficient estimates**	**95% CI lower band**	**95% CI upper band**	**p-value**	**Proportion of mediation**
***Overall*** ^***a***^					
Total association	0.52	0.26	0.77	< 0.001	1
Direct association	0.16	−0.08	0.41	0.18	0.32
Total indirect association	0.35	0.24	0.47	< 0.001	0.68
*Indirect association through:*					
Self-efficacy	0.04	−0.01	0.10	0.15	0.08
Resilience	0.31	0.19	0.43	< 0.001	0.60
***Female*** ^***b***^					
Total association	0.58	0.26	0.9	< 0.001	1
Direct association	0.23	−0.09	0.56	0.16	0.40
Total indirect association	0.35	0.21	0.48	< 0.001	0.60
*Indirect association through:*					
Self-efficacy	0.04	−0.02	0.10	0.25	0.06
Resilience	0.31	0.17	0.45	< 0.001	0.54
***Male*** ^***b***^					
Total association	0.38	−0.04	0.79	0.08	1
Direct association	0.03	−0.34	0.40	0.89	0.07
Total indirect association	0.35	0.15	0.55	< 0.001	0.93
*Indirect association through:*					
Self-efficacy	0.06	−0.05	0.16	0.31	0.15
Resilience	0.29	0.10	0.49	< 0.001	0.78

^a^Estimated in the multiple-mediator path model; ^b^Estimated in the multi-group path model; CI = Confidence interval.

The multi-group path models also had satisfactory goodness-of-fit. The model revealed comparable indirect associations with gender, but the direct associations in males were only 13% of that in females (Table 3). As a result, the total association between PA and mental well-being was only significant in females (b = 0.58, p < 0.001) but not in males (b = 0.38, p = 0.08).

Sensitivity analyses showed our findings were robust for the choice of estimation method and missing data handling.

## Discussion

The findings from this study provided evidence for the association between PA level and mental well-being in Chinese adolescents and supported the psychological hypotheses for this relationship.

Although the study was conducted on adolescents in Hong Kong, the results should be applicable to other major cities in China. Given the rapid economic development in recent decades, the Human Development Index of major Chinese cities such as Shanghai are only 5.9% lower than that of Hong Kong [30]. Reports in the literature also showed a similar social change and socioeconomic disparity in regards to health between Hong Kong and the rest of China [31]. More importantly, a large proportion of Hong Kong’s population is predominantly ethnic Chinese and they share similar cultural backgrounds with those in other major Chinese cities [32].

### Relationship between PA level and mental well-being

The correlation coefficient of 0.66 found between PA and mental well-being of adolescents in our study is equivalent to a large effect size according to Cohen’s criteria [33]. It is interesting to note that this effect size was much larger than that reported in a previous study [9]. In their cross-sectional survey in urban China, the odds ratio of having depression between highly active and sedentary adolescents was 1.66, which is equivalent to a small effect size according to Cohen’s criteria [34]. This discrepancy may be because mental well-being is a much broader concept than a specific mental illness like depression. On the other hand, two studies on university students and middle-aged women identified medium to large effect sizes in mental health related quality of life [35,36], which were consistent with our study. This study demonstrated the quantification of the association between PA and mental health in Chinese populations, which would be useful in cross-cultural comparisons in future meta-analyses.

### Gender differences

In our study, the indirect associations between PA level and mental well-being were comparable in female and male students, but the direct (and thus the total) association was much stronger in females than in males. This was in contrast with the findings from a study in the US, where a significant inverse relationship between mental health service utilisation and accelerometer readings were found in men but not in women [37]. Cultural differences between Western and Chinese populations could lead to large differences in gender patterns in participation of certain PA. For example, a sport or PA regarded as feminine in one culture might be viewed as neutral in other cultures [38]. Such cultural differences in PA participation could in turn moderate the association between PA and mental health. Further epidemiological studies investigating different types of PA (e.g., competitive sports or active transport) would be necessary to examine this issue in more detail.

### From resilience to mental well-being

Resilience was the only significant mediator identified in the association between PA and mental well-being and accounted for 60% of this relationship. The role of resilience in this relationship could be explained by the challenge model. This model proposes a curvilinear relationship between risk (e.g., stressors) and negative outcome (e.g., depression), such that a moderate manageable risk would provide better long-term outcome than no risk or severe unmanageable risks [39]. During PA and sport activities, adolescents would encounter stress related to learning a difficult skill, challenges imposed by competitors, and the frustration of losing [40]. These stressors may impair the adolescent’s mental well-being in the short-term, but they should be able to overcome these with the support of their peers, coaches, and teachers. The process of falling down and climbing up enables adolescents to develop a more resilient mind-set and strengthen their problem-solving skills, which may become valuable assets to help them overcome stresses and difficulties. This could explain why exercising adolescents had better mental health than their sedentary peers [41].

### Insignificance of self-efficacy and social connectedness

Self-efficacy was significant in the single-mediator model but not in the multiple-mediator model, which could be due to the complex nature of resilience that overshadowed the effect of self-efficacy [21]. Even if self-efficacy was a mediator, the current evidence suggest that its effect was much less important than resilience.

Social connectedness has been reported to have associations with PA [10], but we did not identify such a relationship in this study. Insufficient statistical power is unlikely to be a factor as the study sample size had 80% power to detect small effect sizes (r = 0.10) at a 0.05 significance level. Nonetheless, we should not completely disregard the social benefits of PA, as social interactions may only be apparent in well-structured and supervised sporting activities [42].

### Unexplained association between PA and mental well-being

In our final path model, 40% of the association between PA and mental well-being remains unexplained. This remaining portion could be related to biological changes induced by PA, which we did not measure in this study. Exercise has been found to be associated with increased synaptic transmission of monoamines (e.g., serotonin and dopamine), which would have the same mechanism of action as certain antidepressants [12]. Physical exercise could also increase the body’s level of β-endorphins, which in turn enhances mood [12].

### Implications

Our findings demonstrated the significant role of resilience in mediating the positive associations between of PA and mental well-being among Chinese adolescents. Therefore, PA programmes that target resilience enhancement to improve adolescents’ mental health should be promoted. Such interventions should provide adequate opportunities for challenge, which develop the participants’ resilience. On the other hand, it will also be necessary to provide sufficient support to avoid undue stress. The programmes should be well-structured to facilitate resilience building; for example, a debriefing session after the exercise could be included to help adolescents reflect on their performance and develop problem-solving strategies [43]. A number of relevant theoretical frameworks have been developed, such as positive youth development through sports [44].

### Limitations

Our study has several limitations. First, causal relationships could not be established in this cross-sectional study as reverse causation could occur. Second, there may be unobserved confounders that can affect the findings. For example, self-esteem could affect both self-reported PA and mental well-being, which could inflate the association between PA and mental health. However, as the PARCY questionnaire has been validated in local studies with good criterion validity, these factors are unlikely to affect our conclusion. Third, although the current choice of variables should be sufficient to represent the psychological and social hypotheses between PA and mental health, there may still be some potential mediators that we have not considered in this study.

## Conclusions

This is one of the first studies that has verified an association between PA and mental well-being among Chinese adolescents. The findings could provide insights for cross-cultural comparisons between the East and the West. Our results support a psychological but not social hypothesis for explaining the relationship between PA and mental health that showed resilience as the sole significant mediator. These findings suggest that PA interventions focusing on resilience enhancement may improve adolescents’ holistic mental well-being. Future epidemiological studies will be needed to investigate other potential moderators to this relationship, including age and types of PA.
